# Engineering of pentatricopeptide repeat proteins in organellar gene regulation

**DOI:** 10.3389/fpls.2023.1144298

**Published:** 2023-03-01

**Authors:** Kwanuk Lee, Hunseung Kang

**Affiliations:** ^1^ Department of Biology, Jeju National University, Jeju, Republic of Korea; ^2^ Department of Applied Biology, College of Agriculture and Life Sciences, Chonnam National University, Gwangju, Republic of Korea

**Keywords:** engineering PPR proteins, organellar gene expression, organellar RNA metabolism, chloroplast, mitochondria

## Introduction

Plant organelles, including chloroplasts and mitochondria, derived from free-living cyanobacteria and α-proteobacteria (Andersson et al., 2003; Timmis et al., 2004). During the evolution, the massive organellar genes were transferred to the nuclear genome, resulting in only 3-209 proteins are encoded from the current chloroplast or mitochondrial genome (Kleine et al., 2009). The organelles-encoded proteins are crucial for organellar gene expression (OGE), photosynthesis, organellar electron transport chain, cellular metabolism, and ATP production (Maier et al., 2013). Notably, OGE is chiefly regulated at post-transcriptional steps, including RNA cleavage, RNA stability, RNA editing (C to U), and RNA splicing (Lee and Kang, 2016; Lee and Kang, 2020). The fine-tuned regulation of organellar post-transcriptional RNA metabolism essentially require hundreds of the nucleus-encoded chloroplast and mitochondrial RNA-binding proteins (nCMRBPs), including pentatricopeptide repeat (PPR) proteins, chloroplast ribosome maturation and splicing domain (CRM) proteins, DEAD-Box RNA helicases (RHs), S1 domain-containing proteins (SDPs), and mitochondrial transcription termination factors (mTERFs), during the entire period of plant growth and development (Hammani and Giegé, 2014; Quesada, 2016; Leister et al., 2017; Lee and Kang, 2020). Many recent studies have demonstrated that the lack of these proteins leads to the defective RNA metabolsim in plants under various environmental stresses (Lee and Kang, 2020; Qin et al., 2021).

PPR proteins are extensively distributed to plant lineages, containing more than 400 family members, and function as organellar-specific RNA-binding proteins (Schmitz-Linneweber and Small, 2008; Barkan and Small, 2014; Brown et al., 2014). Interestingly, a redesign of PPR tracts was shown to recognize programmed RNA targets and to control organellar gene expression (Coquille et al., 2014; Yagi et al., 2014; Colas des Francs-Small et al., 2018; McDowell et al., 2022). Here, we highlight a novel idea to redesign the PPR tracts of PPR4 and PPR19 proteins, which could affect the affinity of cognate RNAs in plant’s response to developmental and environmental cues.

## Mechanism of P-type PPR proteins for target RNA recognition

The PPR constitutes a pair of antiparallel α helices of degenerate 35-amino acid tandem repeated-motifs ranging from 2 to 30 tracts (Small and Peeters, 2000; Barkan and Small, 2014). The binding specificity of PPR motifs relies on the basis of 1 repeat to 1 nucleotide binding module, leading to the association of single-stranded RNA (Barkan and Small, 2014; Cheng et al., 2016). P-class PPR proteins mainly consist of pure PPR motifs, which are important for organellar transcript processing, RNA stabilization, and group II intron splicing (Barkan and Small, 2014; Cheng et al., 2016; Wang et al., 2021). The PPR-RNA binding codes show that the amino acid residues at the fifth and thirty-fifth positions are essential for sequence specificity in the association of PPR protein-RNA substrate (Barkan and Small, 2014; Coquille et al., 2014; Shen et al., 2015; Yan et al., 2019). Importantly, amino acid codes that recognize the specific RNA bases harbor side chains playing an important role for hydrogen bond interaction with the similar manner of Watson and Crick base paring (Barkan and Small, 2014; Coquille et al., 2014; Shen et al., 2016), suggesting further possibility to predict and/or customize target RNA sequences *via* the combinational PPR-RNA binding code.

## Redesign of artificial PPR proteins in organelles

To date, the usage of amino acid codes for nucleotide recognition suggests that modifying amino acid of PPR motifs causes a weak or strong ability in perceiving target RNA substrates (Barkan et al., 2012; Yin et al., 2013; Shen et al., 2015; Yan et al., 2019). Indeed, artificial PPRs exhibit the ability of recognizing programmed RNA targets and can modulate chloroplast gene expression (Coquille et al., 2014; Yagi et al., 2014). For instance, ZmPPR10 recognizing 5’ UTR of chloroplast Zm*atpH* was modified to successfully switch on the nuclear transgene expression in tobacco plastids and potato amyloplasts (Rojas et al., 2019; Yu et al., 2019). The native PPR proteins of dPPR*^rbcL^* and dPPR*^petL^* were tailored to be associated with the specific sites in the 5’ UTR of rbcL and petL mRNAs in Arabidopsis chloroplasts, respectively (Manavski et al., 2021). The RPF2, an Arabidopsis mitochondrial PPR protein, specifically binds to two cognate RNA sites located within the 5’ UTRs of *cox3* and *nad9* genes (Colas des Francs-Small et al., 2018). Interestingly, the reprogrammed PPR tracts of RFP2 protein, which were designed to bind new target RNA sequences within the open reading frame of *nad6*, recognized the new RNA target site and subsequently led to a cleavage of the RNA molecule *in vivo* (Colas des Francs-Small et al., 2018). These results demonstrate the specificities and *in vivo* functionalities of the artificial PPR proteins.

## Engineering of PPR4 and PPR19 proteins for organellar gene regulation

**Figure 1:**
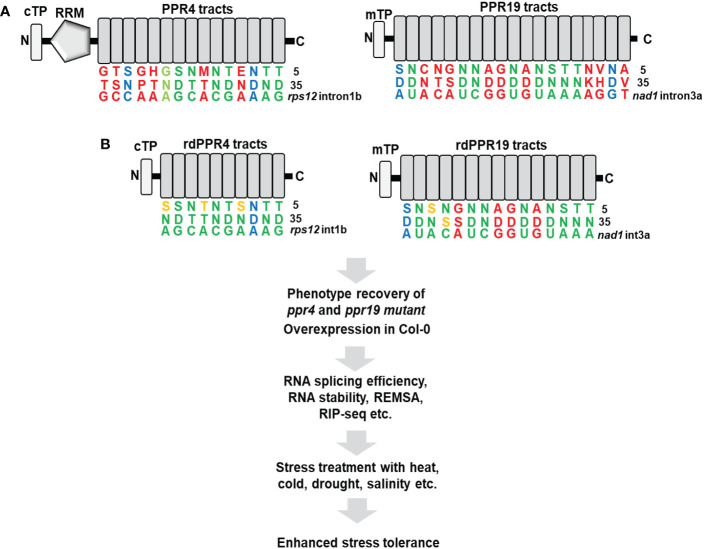
A redesign of Arabidopsis PPR4 and PPR19 proteins targeting to native RNA substrates. **(A)** The combination of the fifth and thirty-fifth amino acids on the native PPR4 and PPR19 proteins specifically recognizes target RNA sequences indicated with green colors for perfect matches, light green colors for less perfect matches, blue colors for mismatches, and red colors for no predictable matches. **(B)** The redesigned Arabidopsis PPR4 and PPR19 proteins with reprogrammed PPR tracts, which is named rdPPR4 tracks and rdPPR19 tracks. The RRM and N-terminal PPR tracts of the PPR4 protein and the C-terminal PPR tracts of the PPR19 protein, which do not participate in target RNA recognition, are eliminated. The PPR-RNA binding codes are modified for better matches with its cognate target RNA sequences as shown with orange colors. REMSA, RNA electrophoretic mobility shift assay; RIP-seq, RNA immunoprecipitation-sequencing; cTP, chloroplast transit peptide; mTP, mitochondrial transit peptide.

Previous studies showed that PPR19, which includes tandem-repeated 19 PPR tracts, is crucial for the stabilization of *nad1* intron 3a in Arabidopsis mitochondria and PPR4, which possesses one RNA-recognition motif (RRM) and tandem-repeated 15 PPR tracts, plays an essential role in the splicing of *rps12* intron 1b in Arabidopsis chloroplasts (Lee et al., 2017; Lee et al., 2019). Although the RRM and PPR motifs of PPR4 are associated with the strong RNA-binding activity of the PPR4 protein, the PPR motifs alone can strongly bind to the specific sequences of *rps12* intron 1b (Lee et al., 2019). Importantly, the designed 11 and 14 PPR tracts showed a similar binding affinity to intended target substrates (Miranda et al., 2018), whereas the longer repeated-tracts tend to promote off-target action and reduce target-binding specificity. Notably, previous researches focused on switching on transgenes in chloroplasts or targeting to new RNA substrates in chloroplasts and mitochondria by combinations of manipulated PPR motifs (Colas des Francs-Small et al., 2018; Miranda et al., 2018; Rojas et al., 2019; Yu et al., 2019; Manavski et al., 2021). However, we suggest that rdPPR4 and rdPPR19 proteins are manipulated in its motifs for promoting the affinity of the proteins to its cognate RNAs (**Figure 1**). We anticipate that these optimizations will enhance the splicing of chloroplast *rps12* intron 1 and the stability of *nad1* intron 3 in native target location, which will increase the function of photosystems and mitochondrial electron transfer chains in plants under optimal conditions and environmental cues. The improvement of organellar functions will confer the phenotypic tolerance in plant’s response to abiotic stresses, including high salinity, drought, extreme temperatures, and high light.

## Conclusion and perspectives

Engineering PPR proteins can be harnessed for the manipulation of intron splicing and/or stabilization of organellar RNA molecules, and these redesigned PPR proteins will be a potential means to improve plant’s fitness to developmental and environmental cues. Engineered PPR proteins can endow the PPR proteins with a scaffolder for RNA targets, which can be potentially applicable for synthetic and RNA biology. Engineering PPR proteins combined with the artificial organelles-trafficking system can be utilized in agricultural crops to produce or restore cytoplasmic male sterility (CMS) which maternally fails to produce fertile pollens due to abnormalities of mitochondrial RNA metabolism.

## Author contributions

All authors listed have made a substantial, direct, and intellectual contribution to the work and approved it for publication.
